# Healthcare-associated foodborne outbreaks in high-income countries: a literature review and surveillance study, 16 OEDC countries, 2001 to 2019

**DOI:** 10.2807/1560-7917.ES.2021.26.41.2001278

**Published:** 2021-10-14

**Authors:** Idesbald Boone, Bettina Rosner, Raskit Lachmann, Michele Luca D'Errico, Luigi Iannetti, Yves Van der Stede, Frank Boelaert, Steen Ethelberg, Tim Eckmanns, Klaus Stark, Sebastian Haller, Hendrik Wilking

**Affiliations:** 1 Robert Koch Institute, Department of Infectious Disease Epidemiology, Berlin, Germany; 2 Istituto Superiore di Sanità, Department of Food Safety, Nutrition and Veterinary Public Health, Rome, Italy; 3 Istituto Zooprofilattico Sperimentale dell'Abruzzo e del Molise G. Caporale, National Reference Laboratory for Listeria monocytogenes, Teramo, Italy; 4 European Food Safety Authority, Parma, Italy; 5 Statens Serum Institut, Infectious Disease Epidemiology and Prevention, Copenhagen, Denmark

**Keywords:** healthcare-associated infections, foodborne outbreaks, Salmonella, Norovirus, Listeria monocytogenes, food hygiene

## Abstract

**Background:**

Healthcare-associated foodborne outbreaks (HA-FBO) may have severe consequences, especially in vulnerable groups.

**Aim:**

The aim was to describe the current state of HA-FBO and propose public health recommendations for prevention.

**Methods:**

We searched PubMed, the Outbreak Database (Charité, University Medicine Berlin), and hand-searched reference lists for HA-FBO with outbreak onset between 2001 and 2018 from Organisation for Economic Co-operation and Development (OECD) countries and HA-FBO (2012–2018) from the German surveillance system. Additionally, data from the European Food Safety Authority were analysed.

**Results:**

The literature search retrieved 57 HA-FBO from 16 OECD countries, primarily in the US (n = 11), Germany (n = 11) and the United Kingdom (n = 9). In addition, 28 HA-FBO were retrieved from the German surveillance system. Based on the number of outbreaks, the top three pathogens associated with the overall 85 HA-FBO were *Salmonella* (n = 24), norovirus (n = 22) and *Listeria monocytogenes* (n = 19). Based on the number of deaths, *L. monocytogenes* was the main pathogen causing HA-FBO. Frequently reported implicated foods were ‘mixed foods’ (n = 16), ‘vegetables and fruits’ (n = 15) and ‘meat and meat products’ (n = 10). Consumption of high-risk food by vulnerable patients, inadequate time-temperature control, insufficient kitchen hygiene and food hygiene and carriers of pathogens among food handlers were reported as reasons for HA-FBO.

**Conclusion:**

To prevent HA-FBO, the supply of high-risk food to vulnerable people should be avoided. Well working outbreak surveillance facilitates early detection and requires close interdisciplinary collaboration and exchange of information between hospitals, food safety and public health authorities.

## Introduction

Yearly, 23 million foodborne disease illnesses and 5,000 deaths are estimated in the World Health Organization (WHO) European Region, and 41 foodborne Disability Adjusted Life Years (DALYs) per 100,000 population were estimated for the WHO Sub-Region EUR A in 2010 [1]. In Europe, a total of 5,146 foodborne and waterborne outbreaks, including 48,365 cases of illness and 40 deaths were reported to the European Food Safety Authority (EFSA) in 2018 [2]. Vulnerable populations, including elderly patients, immunocompromised patients, children younger than five years old and pregnant women are more susceptible to foodborne infections and are more prone to develop severe courses of disease compared with healthy people [3]. Therefore, healthcare is a setting where foodborne outbreaks (FBO) can cause considerable morbidity and mortality. In 2020, 20.6% of the European Union (EU) population was aged 65 years and older [4]. As the proportion of elderly people is projected to further increase, the share of the vulnerable population as patients in healthcare facilities (HCF) is likely to increase and thereby the risk associated with healthcare-associated foodborne outbreaks (HA-FBO). Personnel (medical and non-medical staff, food handlers etc) of HCF may also be at risk for HA-FBO and be a source of further spread in healthcare settings and elsewhere. This can cause major disruption of services [5].

So far, literature reviews have covered pathogens responsible for HA-FBO, including *Salmonella* [6], *Listeria monocytogenes* [7-9] and norovirus [10,11] and focused on microbiological food safety issues in healthcare settings [5,12]. Between 2014 and 2019, a listeriosis outbreak in Germany affected 13 cases who had an inpatient stay in 12 different HCF during the incubation period [13]. In the United Kingdom (UK) in 2019, nine listeriosis cases of which seven died, had consumed sandwiches in seven HCF during the incubation period [14].

We conducted a literature review to describe the causative agents including bacteria, viruses, parasites and fungi, the incriminated food vehicles and other outbreak characteristics of HA-FBO in 37 countries that are members of the Organisation for Economic Cooperation and Development (OECD) [15]. Furthermore, we analysed German surveillance data and data from the EFSA on HA-FBO. The aim of this article is to describe the current status of HA-FBO in order to improve surveillance and provide public health recommendations for prevention.

## Methods

We defined HA-FBO as: two or more cases of the same disease linked to a healthcare facility (HCF) and epidemiologically linked in place, time and likely to the same food source. Healthcare facilities included hospitals, rehabilitation centres, nursing homes and multiple settings (at least two of the previous categories).

We conducted: (i) a literature review in PubMed on publications between January 2001 and 26 April 2019; and a search for outbreak reports on the Outbreak Database published between January 2001 and 26 April 2019; (ii) an analysis of German national surveillance data on HA-FBO between 2012 and 2018 and; (iii) an analysis of data in the EU foodborne outbreak database managed by EFSA between 2010 and 2018.

### Literature review

We included all outbreaks with outbreak onset between 2001 and 2018 that occurred in a HCF in an OECD country [15] and where foodborne transmission was mentioned. Outbreaks were included regardless of study design and without language restrictions. Healthcare staff and workers in the HCF affected in the outbreaks were included in the case numbers. Outbreaks were included regardless of the strength of evidence that could link FBO cases to a food vehicle. 

We excluded descriptions of outbreaks that did not occur in a member country of the Organisation for Economic Co-operation and Development (OECD), community outbreaks, pre-2001 outbreaks, outbreaks that were unlikely foodborne (e.g. person-to-person transmission, contaminated equipment), due to water consumption, involving enteral/parenteral (powdered) formulae, outbreaks with only members of HCF personnel affected, and caused by histamine poisoning, non-original studies (literature reviews).

First, we carried out a search on PubMed on the 26 April 2019 for publications from 2001 onwards. We used search terms, respecting available synonyms for the following categories: healthcare-associated, outbreak, and foodborne pathogens (Supplement S1).

In addition, we conducted a search for outbreak reports in the Outbreak Database, a worldwide database for healthcare-associated outbreaks (www.outbreak-database.com) [16]. This database is managed by the Institute for Hygiene and Environmental Medicine at the Charité, University Medicine Berlin, Germany. Currently it contains 3,682 outbreak reports published between 1956 and 2020 and provides information in different fields and categories, such as geographical setting, demographics of persons affected, pathogen, source, transmission and measures. We queried this database on 26 April 2019 for outbreaks published from 2001 onwards using the search term ‘food’.

Finally, we hand-searched the reference lists of included literature for additional relevant titles. All references were managed in EndNote version X7 (Thomson Reuters, New York, United States (US)).

Titles and abstracts were screened by the first author (IB), followed by a full text screening for eligibility (Figure).

**Figure:**
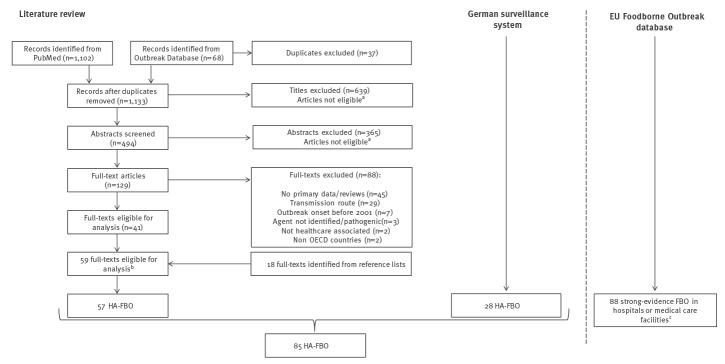
Flowchart showing the data sources to identify healthcare-associated foodborne outbreaks in OECD countries, 2001–2019

ECDC: European Centre for Disease Prevention and Control; EFSA: European Food Safety Authority; EU: European Union; FBO: foodborne outbreaks; HA-FBO: healthcare-associated foodborne outbreaks; OECD: Organisation for Economic Co-operation and Development;

^a^ Not from a member country of the Organisation for Economic Co-operation and Development (OECD), only community outbreak, pre-2001 outbreak, non-food transmission (person-to-person transmission, contaminated equipment, enteral/parenteral (powdered) formulae), waterborne, caused by histamine poisoning, non-original study (literature review).

^b^ Representing 57 HA-FBO, there was one outbreak covered in three publications, one outbreak covered in two outbreaks, and one publication which included two outbreaks.

^c^ One outbreak due to histamine fish poisoning was previously excluded.

### Surveillance data from Germany

The German electronic surveillance system (SurvNet@RKI) covers infectious diseases that are mandatorily notifiable according to Germany’s Protection against Infection Act [17] that came into effect in 2001 and since 2012 a systematic national data collection for the notification of healthcare-associated infection outbreaks is in place [18]. We searched the surveillance system for HA-FBO using the selection criteria ‘outbreaks’ AND ‘foodborne’ AND ‘healthcare facilities’ OR ‘nursing homes’ notified between 2012 and 2018 in Germany. The search date was 11 September 2019. The FBO were categorised as ‘strong-evidence’ and ‘weak-evidence’ according to the EFSA classification which is based on the strength of evidence for the association between illnesses and a suspected food vehicle in the FBO [19]. Information on likely food vehicles was extracted from the available data for ‘strong-evidence’ outbreaks only. For FBO that were classified as ‘weak-evidence’ information on suspected food vehicles was not always available, or the association of illnesses and suspected food vehicles was considered as vague.

### European Union foodborne outbreak database

Since 2004, EFSA has been tasked with the EU-wide data collection on FBO [20]. The EU system for monitoring and collection of information on FBO is based on the Directive 2003/99/EC of the European Parliament and of the Council. The reporting of data on FBO including causative agents and incriminated foodstuffs by the EU countries is mandatory [21]. Data on strong-evidence FBO during the years 2010–2018 in the setting ‘hospital or medical care facilities’ were publicly available as supplemental material complementing the published EU annual summary reports or available on the EFSA Knowledge Junction [22-30].

### Data extraction

We extracted data into a Microsoft Excel template. The following data were systematically extracted: publication characteristics (author(s), publication year, journal), country, outbreak year, healthcare setting, causative agent, number of cases, deaths, patient age and sex distribution if available, suspected food vehicle, associated factors (inadequate time-temperature control, insufficient kitchen hygiene and food hygiene processing, carriers of pathogens among food handlers, consumption of high-risk food items), and microbiological or epidemiological evidence regarding the link between cases and suspected food vehicles (i.e. descriptive or analytical epidemiology, microbiological detection, product-tracing).

The HA-FBO data extracted from the literature review were combined with HA-FBO data from the German surveillance system and merged into one dataset. Data from the EU foodborne outbreak database were analysed separately.

### Data analyses

We calculated total number of outbreaks by pathogen, the number of outbreaks cases (median and range) and the number of deaths stratified by healthcare setting, food vehicles and year of outbreak.

### Ethical statement

Ethical approval was not applicable, as all data were based on published data and anonymously transmitted surveillance data.

### Results

We retrieved a total of 1,133 entries from PubMed and from the Outbreak Database. Additionally, we identified 18 studies from reference lists screening (Figure).

In total 59 publications describing 57 HA-FBO fulfilled the selection criteria (Supplement S1, Table S1). All included publications were in English, except for two publications in German [31,32] and one in Spanish [33]. HA-FBO were reported from 16 OECD countries: Germany [31,32,34-42], US [43-55], UK [56-63], Spain [33,64-66], Japan [67-71], Austria [72-74], Australia [75,76], Canada [44,77,78], Denmark [79,80], France [81,82], Finland [83], Greece [84], Italy [85], Norway [86], the Netherlands [36,87], Turkey [88] and included three multinational outbreaks [36,44,56]. The countries with the highest number of published HA-FBO were Germany (11/57), the US (11/57) and the UK (9/57). Between 2001 and 2018, there was no trend in time, except that no published HA-FBO was reported with outbreak onset in 2015, 2017 and 2018, most likely because of publication delay.

From the German surveillance data (2012–2018), 28 HA-FBO were identified. Of these, nine HA-FBO (32%) had been classified as strong-evidence-FBO (Supplement S1, Table S1), four due to norovirus, two due to *Listeria monocytogenes*, two due to *Salmonella* and one due to *Bacillus cereus*. Nineteen HA-FBO had been classified as weak-evidence FBO (nine due to norovirus, six due to *Salmonella*, two due to *Campylobacter jejuni*, one due to *Bacillus cereus* and one due to *Clostridium perfringens*).

Of the combined 85 HA-FBO in total, 24 were caused by *Salmonella*, 22 (26%) by norovirus and 19 (22%) by *L. monocytogenes* (Table 1). One HA-FBO was caused by the parasitic pathogen *Cyclospora cayetanensis* [55], while another one was associated with the fungus *Blastoschizomyces capitatus* [66]. In total, 3,802 cases were associated with these outbreaks, and 90 deaths occurred (2.4%). Specific details on each HA-FBO, including the type of evidence linking case-patients and suspected food vehicles are listed in the Supplement S1, Table S1.

**Table 1:** Distribution of pathogens in healthcare-associated foodborne outbreaks by healthcare setting, 16 OEDC high-income countries^a^, 2001–2019 (n =85)

Pathogen	Hospital				Nursing home				Rehabilitation centre			Multiple settings				Total Outbreaks
	Outbreaks (n)	Case-patients		Deaths (n)	Outbreaks (n)	Case-patients		Deaths (n)	Outbreaks (n)	Case-patients		Outbreaks (n)	Case-patients		Deaths (n)	
		Median	Range			Median	Range			Media (n)	Range		Media (n)	Range		
*Salmonella^b^*	8	31	3–102	4	9^c^	11	3–111	1	2	64	37–90	5	47	2–130	6	24
*Norovirus*	7	21	5–144	0	6	55	2–126	1	5	25	7–50	4^c^	148	102–570	0	22
*Listeria monocytogenes*	16	4	2–17	29	NA	NA	NA	NA	NA	NA	NA	3	20	18–48	36	19
*Bacillus cereus*	1	NA	2	0	2	NA	26–33	0	1	NA	106	NA	NA	NA	NA	4
*Clostridium perfringens*	1	NA	54	3	3	10	7–90	0	NA	NA	NA	NA	NA	NA	NA	4
*Escherichia coli*	1	NA	4	0	2	NA	46–107	6	NA	NA	NA	1	NA	109	2	4
*Campylobacter jejuni*	1	NA	21	0	NA	NA	NA	NA	2	NA	15	NA	NA	NA	NA	3
Group A streptococci	1	NA	251	0	NA	NA	NA	NA	NA	NA	NA	NA	NA	NA	NA	1
*Klebsiella pneumoniae*	1	NA	35	0	NA	NA	NA	NA	NA	NA	NA	NA	NA	NA	NA	1
*Citrobacter freundii*	1	NA	76^d^	0	NA	NA	NA	NA	NA	NA	NA	NA	NA	NA	NA	1
*Cyclospora cayetanensis*	NA	NA	NA	NA	1	NA	96	0	NA	NA	NA	NA	NA	NA	NA	1
*Blastoschizomyces capitatus*	1	NA	4	2	NA	NA	NA	NA	NA	NA	NA	NA	NA	NA	NA	1
Total	39	8	2–251	38	23	28	2–126	8	10	30	7–106	13	65	2–570	44	85

NA: not applicable; OEDC: Organisation for Economic Co-operation and Development.

^a^ With exception of Turkey which is an upper middle-income OECD country.

^b^ Several zoonotic types: *Salmonella* Enteritidis, *S*. Bovismorbificans, *S*. Brandenburg, *S*. Corvallis, *S*. Derby, *S.* Infantis, *S*. Javiana, *S*. Muenchen, *S*. Newport, *S*. Oranienburg, *S*. Typhimurium.

^c^ For one *Salmonella* HA-FBO [38] and one norovirus HA-FBO [41] numbers of case-patients in healthcare facilities were not provided.

^d^ Colonisations (i.e. not infections).

N: number of HA-FBO, median: median number of outbreak case-patients, Range (min-max) of outbreak case-patients (affected patients/nursing home residents including affected staff) and total number of deaths.

### Case-patients

A high number of case-patients per outbreak was observed in norovirus HA-FBO (median: 35 case-patients, range: 2–570), with one reported death. Salmonellosis HA-FBO included in median 29 case-patients per outbreak (range: 2–130) and altogether 11 deaths were attributed to these outbreaks. In contrast, listeriosis HA-FBO were typically characterised by a smaller number of case-patients per outbreak (median: 5, range: 2–48 case-patients) but a higher number of deaths. In total 65 deaths (on a total of 90 fatalities; 72%) were associated with listeriosis HA-FBO (median case fatality (CF) per outbreak: 33%, range: 0–100). The four *Escherichia coli* HA-FBO [54,70,71,78] had a high number of case-patients per outbreak (median: 77, range: 4–109), including eight deaths and a CF ranging from 0 to 4.7% (median: 2%).

Specific information on the age of the outbreak cases was available for 55 of 85 (65%) HA-FBO (Supplement S1, Table S1). The median age of the case-patients per HA-FBO was 65 years or older for 28 of 52 HA-FBO with available information. Three further HA-FBO reported a mean age of 65 years or older. In particular, the median age of the case-patients was ≥ 65 years in eight of 13 (62%) listeriosis HA-FBO with quantitative information on age available. Eight HA-FBO infants below 1 year of age were affected ([44,70,82], Supplement S1, Table S1). The proportion of female case-patients was 50% (59/117 outbreak cases) in 13 listeriosis HA-FBO, 51% (252/490 outbreak cases) in 14 norovirus HA-FBO, and 59% (216/364 outbreak cases) in 12 salmonellosis HA-FBO. Personnel (medical and non-medical staff, food handlers etc) of HCF were mentioned among the case-patients in 27/61 (43%) HA-FBO.

#### Settings

In total, 39 of 85 HA-FBO occurred in hospitals, followed by 23 of 85 nursing homes, 13 of 85 multiple healthcare settings and 10 of 85 rehabilitation centres (Table 1). In particular, *L. monocytogenes* was responsible for 16 of 39 of the HA-FBO affected hospitals, whereas in nursing homes, nine of 23 HA-FBO were caused by *Salmonella* spp. In some HA-FBO occurring in hospital settings, several wards in the same hospital or even several hospitals were affected [35,42,47,48,76]. In some HA-FBO in the nursing home setting, the outbreak involved several nursing home sites [37,71].

#### Food vehicles

Different food sources were associated with bacterial, viral infections, parasitic or fungal infections. Table 2 shows the top three food categories associated with listeriosis, salmonellosis and norovirus HA-FBO.

**Table 2:** Top three food categories associated with listeriosis, salmonellosis and norovirus healthcare-associated foodborne outbreaks, by number of outbreaks case-patients and deaths, 16 OEDC high-income countries^a^, 2001–2019

Number of outbreaks	Reference^b^	Food categories	Human cases	% Total cases	Deaths	% Total deaths
*Listeria monocytogenes*						
Total			176	NA	65	NA
8	[41,46,48,50,77,80,83,86]	Food of animal origin	132	75.0	50	76.9
8	[57-62]	Mixed food	28	15.9	8	12.3
2	[47]	Vegetables/fruits	13	7.4	6	9.2
*Salmonella*						
Total			916	NA	11	NA
6^c^	[31,45,56,63,87]	Food of animal origin	341	37.2	8	72.7
3	[36,43,44]	Vegetables/fruits	122	13.3	0	0
3	[38,75]	Mixed food	118	12.9	0	0
*Norovirus*						
Total			1,600	NA	1	NA
4^d^	[64,79]	Vegetables/fruits	748	46.8	0	0
2	[72,85]	Mixed food	177	11.1	0	0
2	[33,81]	Food of animal origin	111	6.9	1	100

NA: not applicable; OECD: Organisation for Economic Co-operation and Development.

^a^ With exception of Turkey which is an upper middle-income OECD country.

^b^ Reference: Only references to publications listed; for HA-FBO extracted from the German surveillance system SurvNet, see Supplement S1, Table S1.

^c^ One *Salmonella* Muenchen [38] outbreak associated with raw pork not included (healthcare-associated case numbers not reported).

^d^ One norovirus outbreak [41] associated with frozen strawberries not included (healthcare-associated numbers not reported).

Note: numbers do not add up to n, as only the top three (aggregated) food categories are represented.

Information on the implicated food vehicle was missing or vague in 25 of 85 HA-FBO, including 12 norovirus and nine salmonellosis HA-FBO. Overall the most frequently reported implicated food vehicles in HA-FBO were mixed foods (16/60; 27% of HA-FBO, including seven listeriosis HA-FBO associated with sandwiches) and vegetables and fruits (15/60; 25% of HA-FBOs). In particular, nine HA-FBO have vegetables as likely source of vehicle ([36,42,43,47,54,55,69,71], Supplement S1, Table S1) were associated with a large range of pathogens, whereas four of six HA-FBO associated with fruits were linked to frozen berries contaminated by norovirus ([40,79], Supplement S1, Table S1). ‘Vegetables and fruits’ were the food vehicles associated with the highest number of case-patients (1,308/3,802 case-patients; 35%). Furthermore, meat and meat products were associated with 10/60 (17%) of the HA-FBO. However, the combination of all food vehicles of animal origin accounted for 22 of 60 (37%) HA-FBO. These were mainly associated with *L. monocytogenes* (ready-sliced meat jelly [83], scalded sausage [41], spiced meat roll [80], delicatessen meat [77], milkshake/ice cream [48,51], Camembert cheese [86], and tuna salad [46]), and *Salmonella* spp. (including eggs [45,56,63] and raw pork [37]).

### Antibiotic resistance

In four HA-FBO details on antibiotic-resistant strains were provided. These comprised a HA-FBO caused by a carbapenemase-expressing (VIM-type) *Citrobacter* *freundii* with 76 colonisations in several wards of a university hospital [42], a *S.* Enteritidis outbreak (resistant to nalidixic acid, low level susceptibility to ciprofloxacin) associated with eggs [63], an ESBL-producing *Klebsiella pneumoniae* (SHV1 and CTX-M-15) outbreak (resistant to penicillin and third-generations cephalosporin) affecting patients with indwelling catheter, surgical infections and primary bacteraemia [65] and an ESBL-producing *E. coli* outbreak transmitted through donor breast milk in a neonatal intensive care unit [70].

### Preventable causes

Associated factors that may have contributed to the respective outbreaks were described for 35 of 85 (41%) HA-FBO. Inadequate time or temperature control during food preparation was reported for 16 of the 35 (46%) HA-FBO associated with *Salmonella* [34-36,63,87], *L. monocytogenes* [57,59-62], *C. perfringens* [52,69], *E. coli* [70], *C. jejuni* [74], *C. freundii* [42]. In 10 of 35 (29%) HA-FBO, food or ingredients that were considered risky for vulnerable populations in HCF were mentioned. These included raw pork products [37], deli-meat [77], (uncooked) frozen berries [40,79], raw unpasteurised shell eggs [45,63], raw oysters [81], and bean sprouts [36]. Carriers among kitchen personnel or food handlers were reported for eight of 35 (23%) HA-FBO, including outbreaks associated with *Salmonella* [32,38,43], norovirus [72] and *K. pneumoniae* [65]. Insufficient hygiene practices in processing raw foodstuff or inadequate cleaning of the kitchen, equipment or environment were reported in five HA-FBO, caused by *Salmonella* [38,84], *L. monocytogenes* [50,51] and *K. pneumoniae* [65]. In 29 HA-FBO (16 *L. monocytogenes*, seven *Salmonella*, three norovirus, one *B. cereus*, one *E. coli* and one *Cyclospora cayetanensis* HA-FBO), trace-back investigations linked the HA-FBO to caterer companies or suppliers. Trace-back investigations were most frequently reported for listeriosis [41,47,48,51,57-62,71,76,77,80,83,86], followed by salmonellosis HA-FBO [31,34-36,56,63,75]. In norovirus HA-FBO associated with frozen berries [40,79], an incriminated batch or a company could be identified.

### Healthcare-associated foodborne outbreaks in the European Union foodborne outbreak database

A total of 88 strong-evidence FBO and no waterborne outbreak were reported in hospitals or medical care facilities by 14 EU countries between 2010 and 2018 [22-30]. The majority of these outbreaks were notified by Poland (33/88; 38%) and by France (25/88; 28%). The top three pathogens causing these outbreaks were norovirus (20/88; 23%), *Salmonella* (12/88; 14%) and *C. perfringens* (12/88; 14%). Listeriosis HA-FBO were less frequently reported (8/88; 9%). From these eight strong-evidence listeriosis HA-FBO, 45 illnesses, including seven deaths (CF: 16%) were reported. A single HA-FBO caused by *Trichinella spiralis* with four case-patients was reported by Poland. Distribution of FBO pathogens differed by reporting countries. For instance, 11 of 12 outbreaks due to *C. perfringens* were reported by France. Implicated food vehicles were mainly mixed foods (25/88; 28%), meat and meat products (24/88; 27%) and fruits and vegetables (11/88; 13%). From 14 HA-FBO reports [35-37,39,40,42,56,61,62,73,80-83] that occurred in the EU between 2010 and 2018 we retrieved three matching records of *Listeria* outbreaks in the EU foodborne outbreak database [61,62,83].

## Discussion

The top three pathogens causing HA-FBO retrieved from the published reports and the German surveillance system were *Salmonella*, norovirus, and *L. monocytogenes*, whereas in the EFSA foodborne outbreak database, the top three pathogens causing HA-FBO consisted of norovirus, *Salmonella* and *C. perfringens*. 

In the current study, *Listeria* *monocytogenes* was responsible for the majority of the HA-FBO in the hospital setting and characterised by a high case fatality affecting mainly vulnerable patients with comorbidities and elderly patients. Listeriosis outbreaks were less frequently reported to EFSA’s foodborne outbreak database compared with those included in the current study. In total, 4 of 85 (5%) HA-FBO due to *C. perfringens* were included in this study. A few HA-FBO were caused by less common foodborne pathogens such as *K. pneumoniae* [65], *C. freundii* [42], *Cyclospora cayetanesis* [55] and *B. capitatus* [66] which highlights that such pathogens should not be neglected as HA-FBO. Strong-evidence FBO caused by *Trichinella*, *Cryptosporidium*, *Giardia* and *Anisakis* have been reported to EFSA in different settings between 2010 and 2018, but only one FBO caused by *T. spiralis* was reported in a hospital or medical care facility. Although *Toxoplasma gondii* is prevalent in humans and animals and reporting of foodborne toxoplasmosis disease outbreaks in humans is mandatory in the EU, no FBO caused by this parasite has been reported to EFSA since the start of the FBO reporting, in 2004 [2].

Four HA-FBO resulted in foodborne transmission of antibiotic-resistant strains which is especially relevant in a medical setting [42,63,65,70]. The vehicles were an egg delivery, pre-sliced vegetables, breast milk and in one instance the food item remained unclear. Contaminations were most likely in-house.

In several HA-FBO, several HCF, wards and multiple healthcare settings were affected [76,31,35,36,42,44,47,48,68,77-80,83,87,89]. Moreover, in several HA-FBO, there were outbreak cases in the community in addition to the outbreak cases in the HCF. This implies that different HCFs may have been served by the same kitchen, that caterers provided food to several healthcare institutions and to the community and that large food distribution chains play an important role in HA-FBO. This highlights the importance for food chain distribution analyses of HCFs in outbreak investigations to support laboratory and epidemiological investigations. Food suppliers or caterers for HCF should reinforce their companies’ own checks to guarantee that no contaminated products enter HCF.

Although no retrieved *Listeria* HA-FBO occurred only in nursing homes, three long-term care facilities were affected as part of multiple settings [77,80,83]. Listeriosis HA-FBO are difficult to detect as only single but severe cases occur over a prolonged time. The foodborne aetiology of the bacteraemia is often not detected.

A major part of the HA-FBO was related to mixed food, fruits and vegetables and meat and meat products. The risk of ready-to-eat sandwiches (mixed food) for hospital patients has been highlighted in a listeriosis outbreak in 2019 in the UK [14]. The importance of mixed foods suggests the need for improved hygienic standards for food preparation processing and preparation of food in HCF and external companies delivering ready-to-eat (RTE) meals to HCF, especially RTE that did not receive a treatment to inactivate foodborne pathogens. With respect to HA-FBO caused by fruits and vegetables, frozen berries should be heat-treated before offered for consumption and berries that have not been heated should not be served to vulnerable or immunocompromised patients. Furthermore, fresh produce (e.g. mung bean sprouts [36], raw celery [47], raw spinach [54]) should be considered as a risk for vulnerable patients. Similar to the currents study, the top three HA-FBO reported to EFSA were associated with mixed foods, meat/meat products and vegetables/fruits [22-30].

Some HCF might focus on nutritional risk in vulnerable patients or might be more concerned about diet composition and neglect the microbial food safety and respective guidelines [90]. A diet should therefore be selected that is suitably nutritious and palatable without using high-risk foods. Limited budgets of healthcare institutions may lead to minimising catering costs, which may have an impact on the quality of the food served. In Germany, hospitals spent on average 5.14 Euros on food products per patient per day in 2018 [91]. In Italy in 2016, the average expense for meals in public hospitals was 7.90 Euros per patient per day [92]. In a large proportion of the HA-FBO, both patients and nursing home residents, and staff members including healthcare workers and kitchen personnel were affected, which highlights the role of personnel (medical and non-medical staff, food handlers etc) of HCF in spreading outbreaks, e.g. through further secondary cases, and the disruptive impact on the management of the services of the HCF [3].

Food business operators should improve and constantly control all food production procedures based on the HACCP principles (Regulation (EC) No 852/2004, Hygiene Package) [93], especially when the catering is outsourced. Food safety in HCF should be ensured and supply chains strictly controlled [12]. In Germany, food safety recommendations for healthcare settings are available for the food safety sector [94,95], and the public health and hospital infection control sector [96-98] but can be further elaborated to achieve awareness in practice in more HCF.

With HA-FBO mainly from Germany, the US, and the UK and mainly published in English, we suspect that there was a publication bias which may limit the overall body of evidence on HA-FBOs. We did not include a separate search in national public health journals in local languages, therefore we may have overlooked reports of HA-FBOs. In addition, there may be a lack of systematic surveillance of FBO in HCF as compared with other healthcare-associated infections. The identification of HA-FBO cases in HCF could be complicated among patients with comorbidities. The number of fatalities among patients with comorbidities may have been overestimated, because the foodborne infection may not have been causative for or contributed to the death.

Retrieved HA-FBO did not all provide details on age and sex of the outbreak-cases, settings and description of the cases among healthcare workers and other personal.

No overlaps were found between HA-FBO obtained from the literature review and those retrieved from the German surveillance system, either because these outbreaks were not categorised in a HCF setting, or because the outbreak was not reported in the surveillance system.

Comparisons of the HA-FBO from the current review with the EFSA data on strong-evidence FBO in hospitals or medical care facilities should be made with caution. The low degree of matching between HA-FBO from the literature and the EFSA database can partly be explained by the fact that from the latter only the ‘hospital or medical care facility’ setting was included, and non-matching HA-FBO from the literature may have been classified under different settings in the EFSA database, such as ‘residential institution (nursing home or prison or boarding school)’, ‘disseminated cases’, or ‘multiple places of exposure’.

The fact that *C. perfringens* was the third most reported agent for strong-evidence FBO in setting hospital or medical care facility in the EFSA database may in major part be related to the MS' specific reporting. Noteworthy, the variable place of exposure is an optional data element. More generally, although the data reporting rules follow the same EFSA standard specifications, the surveillance activities of FBO are not fully harmonised across EU countries and differences in sensitivity and type of outbreaks under surveillance exist [99]. For this reason, the difference in the numbers and types of reported FBO, as well as in the causative agents and the type of outbreaks may not necessarily reflect the level of food safety in the countries.

## Conclusion

HCF should ensure that foods, especially ready-to-eat foods offered to patients and nursing home residents are free of pathogens and that high-risk foods are avoided, by implementing food safety measures and strictly controlling food supply chains.

Despite regulations to govern food safety and existing guidelines on food safety, there is still a need to raise awareness including regular food safety training programmes for HCF staff and to enforce adherence to safe food policies, especially regarding food for highly vulnerable or immunocompromised patients. Additionally, in-house contamination of food during preparation should be avoided. Surveillance of healthcare-associated infections should be integrated with surveillance of foodborne diseases to improve detection of HA-FBO. In addition, close interdisciplinary collaboration and exchange of information between hospital infection control, food safety, and public health authorities is necessary.

More studies are necessary to appraise the burden of HA-FBO and more detailed descriptions of HA-FBO including the role of personnel (medical and non-medical staff, food handlers etc) of HCF should be documented in reports and in surveillance data.

## Supplementary Data

266000 Supplement
