# Erratum for Euro Surveill. 2021;26(41)

**DOI:** 10.2807/1560-7917.ES.2021.26.41.2001278e1

**Published:** 2021-10-21

**Authors:** Eurosurveillance editorial team

**Affiliations:** 1 European Centre for Disease Prevention and Control (ECDC), Stockholm, Sweden

In the article ‘Healthcare-associated foodborne outbreaks in high-income countries: a literature review and surveillance study, 16 OECD countries, 2001 to 2019’ by Boone et al. published on 14 October 2021, the following corrections were made to the title: ‘OEDC’ was replaced with ‘OECD’. The same correction was undertaken for the Table 1 title and the respective table note and for the Table 2 title. The errors were corrected on 21 October 2021. We apologise for any inconvenience this may have caused.

