# Supplementary material for: Healthcare-associated foodborne outbreaks in high-income countries: a literature review and surveillance study, 16 OEDC countries, 2001 to 2019
Source: Euro Surveill. 2021 Oct 14;26(41):2001278. doi: 10.2807/1560-7917.ES.2021.26.41.2001278 (PMC8518306; doi:10.2807/1560-7917.ES.2021.26.41.2001278)
Supplement: Supplement [file 20-01278_BOONE_Supplement.pdf]

Supplement S1

Disclaimer
This supplementary material is hosted by Eurosurveillance as supporting information alongside the article [Healthcare-associated foodborne outbreaks in high-income countries: a literature review and surveillance study, 16 OEDC countries, 2001 to 2019], on behalf of the authors, who remain responsible for the accuracy and appropriateness of the content. The same standards for ethics, copyright, attributions and permissions as for the article apply. Supplements are not edited by Eurosurveillance and the journal is not responsible for the maintenance of any links or email addresses provided therein.

Search strategy in PubMed for the literature review of healthcare-associated foodborne outbreaks

((((nosocomial[Title/Abstract] OR "healthcare-associated"[Title/Abstract] OR "healthcare associated"[Title/Abstract] OR "health care associated"[Title/Abstract] OR "health care acquired"[Title/Abstract] OR "healthcare acquired infection"[Title/Abstract] OR HAI[Title/Abstract] OR HCAI[Title/Abstract] OR "hospital"[Title/Abstract] OR clinic[Title/Abstract] OR "health care facility"[Title/Abstract] OR "healthcare facility"[Title/Abstract] OR "health care facilities"[Title/Abstract] OR "health care facilities"[Title/Abstract] OR hospitals[Title/Abstract] OR "rehabilitation center"[Title/Abstract] OR "rehabilitation centers"[Title/Abstract] OR "rehabilitation centre"[Title/Abstract] OR "rehabilitation centres"[Title/Abstract] OR "medical center"[Title/Abstract] OR "medical centers"[Title/Abstract] OR "medical centre"[Title/Abstract] OR "medical centres" [Title/Abstract] OR "medical institution"[Title/Abstract] OR "medical institutions"[Title/Abstract] OR "nursing home"[Title/Abstract] OR "nursing homes"[Title/Abstract] OR "nursery home"[Title/Abstract] OR "nursery homes"[Title/Abstract])) AND (outbreak*[Title/Abstract])) AND (food[Title/Abstract] OR foodborne[Title/Abstract] OR food-borne[Title/Abstract] OR Salmonella[Title/Abstract] OR Campylobacter[Title/Abstract] OR "Bacillus cereus"[Title/Abstract] OR Escherichia[Title/Abstract] OR Listeria[Title/Abstract] OR Norovirus[Title/Abstract])) AND ("2001"[Date - Publication] : "3000"[Date - Publication])

Supplementary Table S1. Overview of published articles on hospital associated foodborne outbreaks (2001-2018, n=57) in OECD countries and HA-FBOs from the German surveillance system (SurvNet, 2012-2018, n=28)

Agent causing outbreak	Country	Likely source or vehicle	Year of outbreak	Healthcare Setting	Evidence: link cases and food vehicle	No of cases	No of deaths	Person-nel in cases	Age groups	Median (range) years	No of female cases/N	% females	Ref [a]
***Salmonella* spp.**													
S. Brandenburg + *S.* Corvallis	Japan	Unknown	2001	Hospital	Epi descriptive; Mibi[b]: detection in food vehicle or its component	8	0	no	Elderly	(66-88)	NA[c]	NA	[1]
S. Enteritidis	Netherlands	Bavarian cream-dessert	2001	Hospital and nursing home	Epi analytical: cohort study	82	5	yes	Elderly	NA	NA	NA	[2]
S. Typhimurium	Australia	Rice pudding, meat based, potato pie	2001	Aged care facility	Epi descriptive; detection in food vehicle or its component; product-tracing	18	0	yes	Adults, Elderly	NA	NA	NA	[3]
S. Enteritidis	UK	Imported eggs	2002	Hospital	Epi descriptive; Mibi: detection in food vehicle or its component	29	1	no	NA	NA	NA	NA	[4]
S. Infantis	Germany	Pasta, vegetables, rice	2002-2009	Rehabilitation oncology clinic	Epi descriptive; Mibi: detection in food vehicle or its component; detection in food chain or its environment	90	0	yes	NA	NA	NA	NA	[5]
S. Javiana	USA	Salad, fresh vegetable	2003	Tertiary hospital. Children's Hospital	Epi analytical: case-control study	101	0	yes	Adults, Children	NA	NA	NA	[6]
S. Enteritidis	Germany	Contaminated pudding/curd dumpling	2004	Hospitals, Elderly homes	Epi analytical: case-control study; Product-tracing	47	0	yes	Adults, Elderly	83 (57-102)[d]	27/37[d]	73	[7]
S. Enteritidis	Greece	Unknown	2005	Hospital	Epi analytical: case-control study	102	2	yes	Adults, Elderly, Children	60[e]	NA	67	[8]
S. Enteritidis	Germany	Bakery products (cake)	2006	Nursing home	Epi analytical: cohort study; Mibi: detection in	111	1	yes	Adults, Elderly	NA	NA	NA	[9]

Agent causing outbreak	Country	Likely source or vehicle	Year of outbreak	Healthcare Setting	Evidence: link cases and food vehicle	No of cases	No of deaths	Person-nel in cases	Age groups	Median (range) years	No of female cases/N	% females	Ref [a]
					food vehicle or its component								
S. Oranienburg	USA + Canada	Fruit salad	2006	Health care facilities	Epi analytical: case-control study	29	0	yes	Adults, Elderly, Infant[f]	59 (8 months-96)[g]	28/41	68[g]	[10]
S. Enteritidis	Germany	Unknown	2007	Hospital	Epi analytical: case-control study	33	0	yes	Adults, Elderly, Children	66 (1-87)	17	61	[11]
S. Enteritidis	Germany	Cheesecake	2010	Tertiary care hospital/children's hospital (3 hospitals)	Epi descriptive	4	0	no	Adults, Children	NA	NA	NA	[12]
S. Newport	Germany + Netherlands	Mung bean sprouts	2011	Rehabilitation clinic in Germany, Hospital in the Netherlands	Epi analytical: case-control study; Mibi: detection in food vehicle or its component; Product-tracing	≥ 2 (2 in NL; NA for HCF in DE)	0	NA	NA	NA	NA	NA	[13]
S. Enteritidis	USA	Raw unpasteurized shell egg	2012	Long-term care facility	Epi analytical: cohort study	21	0	yes	Adults, Elderly	83 (21-94)	15	71	[14]
S. Bovismorbificans	Germany	Unknown	2013	Rehabilitation centre	No details/unknown/weak	37	0	NA	Adults, Children Infants	12 (0-44)	20	46	SN1
S. Derby	Germany	Spreadable raw sausages (Teewurst)	2013-2014	Hospitals and residential institutions	Epi descriptive; Mibi: detection in food vehicle or its component; detection in food chain or its environment	130[h]	1	yes[i]	Adults, Elderly, Children Infants	76 (0-100)[j]	76[j]/150	51[j]	SN2*
S. Enteritidis	Germany	Unknown	2013	Nursing home	No details/unknown/weak	11	0	NA	Adults, Elderly, Children	79 (16-96)	11	100	SN3
S. Enteritidis	UK (part of multinational outbreak)	Eggs from a German producer	2014	Hospital	Epi descriptive; detection in food vehicle or its component; product-tracing	32	1	yes	NA	NA	NA	NA	[15]

Agent causing outbreak	Country	Likely source or vehicle	Year of outbreak	Healthcare Setting	Evidence: link cases and food vehicle	No of cases	No of deaths	Person-nel in cases	Age groups	Median (range) years	No of female cases/N	% females	Ref [a]
S. Enteritidis	Germany	Unknown	2014	Nursing home	No details/unknown/weak	4	0	no	Elderly	88 (85-94)	4	100	SN4
S. Gruppe B	Germany	Unknown	2014	Hospital	No details/unknown/weak	3	0	NA	Adults	31 (24-37)	0	0	SN5
S. Muenchen	Germany	Raw pork products	2014	3 Nursing homes	Epi analytical; Mibi: detection in food vehicle or its component; detection in food chain or its environment	NA	NA	yes ≥ 6	NA	NA	NA	NA	[16]
S. Gruppe B	Germany	Unknown	2015	Nursing home	No details/unknown/weak	9	0	NA	Adults, Elderly, Children	84 (16-98)	8	67	SN6
S. Enteritidis	Germany	Sauce for cabbage roulade	2017	Nursing home	Epi descriptive; Mibi: detection in food vehicle or its component	10	0	no	Elderly	83 (75-93)	10	67	SN7 *
S. Enteritidis	Germany	Unknown	2018	Nursing home	No details/unknown/weak	3	0	NA	Adults, Elderly	56 (55-68)	NA	NA	SN8
**Norovirus**													
Norovirus	Spain	Whipped cream mousse	2001	Elderly home	Epi analytical: case-control study	56	0	yes	Adults, Elderly	mean 83 (56-101)	32	78	[17]
Norovirus[k]	Spain	Dried fruit, strawberry jam	2002	Health Centre	Epi analytical: cohort study	50	0	yes	Adults	NA	NA	NA	[18]
Norovirus	Denmark	Frozen raspberry pieces	2005	Hospital, Nursing homes	Epi analytical: case-control study; cohort study	570	0	NA	Adults, Elderly	NA	NA	NA	[19]
Norovirus	Italy	Thin soup	2006	Elderly home	Epi analytical: cohort study	29	0	yes	Adults, Elderly	74 (19-97)[l]	NA	NA	[20]
Norovirus	Japan	Unknown	2007	Hospital, Long-Term Care facility, Daycare users	Epi analytical: cohort study	102	0	yes	Adults, Elderly	56 (IQR 45-66)	18	33	[21]
Norovirus	Austria	Cold sliced sausage, meat dish with salad, hand-	2009	Healthcare facility (Hospital+rehabilitation	Epi analytical: cohort study, others	148	0	yes	Adults, Elderly?	52[m]	NA	Sex ratio (male	[22]

Agent causing outbreak	Country	Likely source or vehicle	Year of outbreak	Healthcare Setting	Evidence: link cases and food vehicle	No of cases	No of deaths	Person-nel in cases	Age groups	Median (range) years	No of female cases/N	% females	Ref [a]
		rolled spinach pancake		centre, convalescent home)								:female): 0.77[m]	
Norovirus	Germany	Unknown	2011	University hospital	Epi descriptive	144	0	yes	NA	NA	NA	NA	[23]
Norovirus	Germany	Frozen strawberry	2012	Disability care (3), elderly homes (2) rehabilitation clinic (1)	Epi analytical: case-control study; Product-tracing	NA	0	NA	NA	NA	NA	NA	[24]
Norovirus	France	Oysters	2012	Nursing home	Epi analytical: cohort study; Mibi: detection in food vehicle or its component	55	1	yes	Adults, Elderly	NA	NA	NA	[25]
Norovirus	Germany	Unknown	2013	Hospital	No details/unknown/weak	5	0	NA	Adults	35 (29-45)	3	60	SN9
Norovirus	Germany	Unknown dishes prepared in the hospital kitchen	2013	Hospital/medical care facility	Epi descriptive	14	0	NA[l]	Adults, Elderly	72 (23-80)	4	29	SN10*
Norovirus	Germany	Unknown	2016	Rehabilitation centre	No details/unknown/weak	35	0	NA	Adults, Children	7 (1-51)	25	71	SN11
Norovirus	Germany	Raspberry fruit quark, with frozen raspberries[n]	2016	Hospital/medical care facility	Epi descriptive; Mibi: detection in food vehicle or its component; Product-tracing	103	0	NA	Adults, Elderly	34 (19-68)	7	7	SN12*
Norovirus	Germany	Frozen raspberries[o]	2016	Hospital/medical care facility	Epi descriptive; Mibi: detection in food vehicle or its component; detection in food chain or its environment	25	0	NA	Elderly, Adults, Infants	65 (0-79)	6/18[p]	33	SN13*
Norovirus	Germany	Unknown	2016	Hospital	No details/unknown/weak	6	0	NA	Adults, Elderly	76 (40-82)	6	100	SN14

Agent causing outbreak	Country	Likely source or vehicle	Year of outbreak	Healthcare Setting	Evidence: link cases and food vehicle	No of cases	No of deaths	Person-nel in cases	Age groups	Median (range) years	No of female cases/N	% females	Ref [a]
Norovirus	Germany	Unknown	2016	Nursing home	No details/unknown/weak	54	0	NA	Elderly, Adults, Infants	64 (0-93)	4/6[q]	67	SN15
Norovirus	Germany	Unknown	2017	Hospital	No details/unknown/weak	35	0	NA	Adults (80% missing)	NA	25	71	SN16
Norovirus	Germany	Unknown	2017	Hospital	No details/unknown/weak	21	0	NA	Adults, Elderly	48 (18-84)	15	71	SN17
Norovirus	Germany	Unknown	2017	Hospital	No details/unknown/weak	13	0	NA	Adults, Elderly	72 (50-81)	4	31	SN18
Norovirus	Germany	Bread, bread rolls	2017	Nursing home	Epi descriptive	126	0	NA[i]	Elderly, Adults, Infants	69 (0-95)	94	75	SN19*
Norovirus	Germany	Unknown	2018	Rehabilitation centre	No details/unknown/weak	7	0	NA	Adults, Children	24 (1-59)	9	75	SN20
Norovirus	Germany	Unknown	2018	Nursing home	No details/unknown/weak	2	0	no	Elderly	(83-89)	NA	NA	SN21
Listeria monocytogenes													
L. monocytogenes	UK	Sandwiches	2003	Hospital - oncology unit	Epi descriptive; Mibi: detection in food vehicle or its component; detection in food chain or its environment	2	2	no	Adults	(46-63)	2	100	[26]
L. monocytogenes	UK	Prepacked sandwiches	2003	Hospital	Epi descriptive; Mibi: detection in food vehicle or its component	4	0	no	Adults (pregnant)	NA	4	100	[27]
L. monocytogenes	UK	Sandwiches: various fillings	2004	Hospital	Epi descriptive; Mibi: detection in food vehicle or its component; detection in food chain or its environment	2	1	no	Elderly	NA	0	0	[28]

**Agent causing outbreak**	**Country**	**Likely source or vehicle**	**Year of outbreak**	**Healthcare Setting**	**Evidence: link cases and food vehicle**	**No of cases**	**No of deaths**	**Person-nel in cases**	**Age groups**	**Median (range) years**	**No of fema le case s/N**	**% fema les**	**Ref** [a]
L. monocytogenes	Germany	Ready to eat scalded sausage	2006-2007	Hospital	Epi descriptive; Mibi: detection in food vehicle or its component; detection in food chain or its environment	11	5	no	Adults, Elderly	72 (50-83)	4	36	[29]
L. monocytogenes	Norway	Camembert cheese made from pasteurised milk	2007	Tertiary care hospital	Epi descriptive; Mibi: detection in food vehicle or its component	17	3	yes	Adults, Elderly	64 (27-84)	11	65	[30]
L. monocytogenes	Canada	Delicatessen meat	2008	Long-term care facility, hospital	Epi descriptive; Mibi: detection in food vehicle or its component; detection in food chain or its environment; Product-tracing	48	24[r]	no	Adults, Elderly	78 (29-98)	NA	NA	[31]
L. monocytogenes	UK	Sandwiches: various fillings	2008	Hospital	Epi descriptive; Mibi: detection in food vehicle or its component	3	1	no	Elderly	NA	NA	NA	[28]
L. monocytogenes	UK	Sandwiches: various fillings	2008	2 hospitals	Epi descriptive; Mibi: detection in food vehicle or its component; detection in food chain or its environment	7	3	no	> 60 years	67	2	29	[32]
L. monocytogenes	USA	Tuna salad	2008	Hospital	Epi descriptive; Mibi: detection in food vehicle or its component; detection in food chain or its environment	5	3	no	Adults, Elderly	62 (52-92)	1	20	[33]
L. monocytogenes	UK	Sandwiches: various fillings	2010	Hospital	Epi descriptive; Mibi: detection in food vehicle or its component; detection in food chain or its environment	5	1	no	NA	NA	NA	NA	[34]

Agent causing outbreak	Country	Likely source or vehicle	Year of outbreak	Healthcare Setting	Evidence: link cases and food vehicle	No of cases	No of deaths	Person-nel in cases	Age groups	Median (range) years	No of female cases/N	% females	Ref [a]
L. monocytogenes	USA	Diced celery	2010	5 Acute care hospitals	Epi descriptive; Mibi: detection in food chain or its environment	10	5	no	Adults, Elderly	80 (56-93)	5	50	[35]
L. monocytogenes	USA	Milkshake	2010-2015	Hospitals in 2 states	Epi descriptive; Mibi: detection in food vehicle or its component	10	3	no	NA	NA	NA	NA	[36]
L. monocytogenes	UK	Sandwiches: various fillings	2011	Hospital	Epi descriptive	3	0	no	Adults, Elderly	50~59	1	33	[37]
L. monocytogenes	Finland	Ready-sliced meat jelly	2012	Municipal hospital, institutional care	Epi analytical: cohort study; Mibi: detection in food chain or its environment	18 (10 hospital, 8 other HCF	3	no	Elderly	82 in hospital; 73 in other HCF	4/10 in hospital	40 in hospital	[38]
L. monocytogenes	Australia	Dessert	2013	3 Tertiary hospitals	Epi descriptive; Mibi: detection in food chain or its environment	3	1	no	Adults, Elderly	79 (34-82)	NA	NA	[39]
L. monocytogenes	Germany	Mixed salad[s]	2013	Hospital/medical care facility	Mibi: detection in food vehicle or its component; detection in food chain or its environment	3	1	no	Elderly	NA	NA	NA	SN22*
L. monocytogenes	Denmark	Spiced meat roll	2013-2014	Hospitals, long-term care facilities	Epi descriptive; Mibi: detection in food vehicle or its component; detection in food chain or its environment; Product-tracing	20[t] (5+15)	9[u] (1+8)	no	Adults, Elderly	71 (43-90)[v]	23/41[q]	56[q]	[40]
L. monocytogenes	USA	Ice cream mix, milkshake	2014-2015	Hospital	Epi descriptive; Mibi: detection in food vehicle or its component; detection in food chain or its environment	3	0	no	Adults	2 patients 50ies, 1 patient 40ies	1	33	[41-43]
L. monocytogenes	Germany	Iceberg lettuce with yoghurt dressing, Gouda cheese	2014	Hospital/medical care facility	Epi descriptive	2	0	no	Elderly	83 (79-87)	1	50	SN23*

Agent causing outbreak	Country	Likely source or vehicle	Year of outbreak	Healthcare Setting	Evidence: link cases and food vehicle	No of cases	No of deaths	Person-nel in cases	Age groups	Median (range) years	No of female cases/N	% females	Ref [a]
Bacillus cereus	France	Pooled breast milk	2010	Tertiary care teaching hospital	Epi descriptive	2	0	no	Newborn	(29-30 weeks)	NA	NA	[44]
B. cereus	Austria	Fruit salad and deer ragout	2013	Rehabilitation clinic	Epi analytical: cohort study; Mibi: detection in food vehicle or its component	106	0	no	Adults, Elderly?	59	NA	Sex ratio (male:female ratio) 0.74: 1	[45]
B. cereus	Germany	Unknown	2017	Nursing home	No details/unknown/weak	33	0	no	Adults, Elderly	86 (63-97)	26	79	SN24
B. cereus	Germany	Canapes with turkey, canapes with ham, salmon canapes, vital wraps with vegetables	2017	Nursing home	Epi descriptive; Mibi: detection in food vehicle or its component; Product-tracing	26	0	yes	Adults, Elderly	70 (54-87)	3	75	SN25*
Clostridium perfringens	Japan	Boiled beans	2001	Nursing home	Epi descriptive	90	0	no	Elderly	NA	NA	NA	[46]
C. perfringens	USA	Meal with turkey	2001	Residential care facility for mentally ill	Epi descriptive	7	0	no	Adults	48 (30-59)	4	57	[47]
C. perfringens	USA	Chicken	2010	Psychiatric hospital	Epi descriptive; Mibi: detection in food vehicle or its component	54	3	yes	Adults, Elderly?	NA	NA	NA	[48]
C. perfringens	Germany	Unknown	2015	Nursing home	No details/unknown/weak	10	0	NA	Adults, Elderly	86 (46-100)	6	60	SN26
Escherichia coli O157:H7	Canada	Salads and sandwiches	2002	Psychiatric hospital, health care facilities	Epi analytical: case-control study	109	2	yes	Adults	NA	NA	NA	[49]
E. coli O157:H7	USA	Raw spinach	2003	Nursing home	Epi analytical: case-control study	46	1	yes	Elderly	NA	NA	NA	[50]

Agent causing outbreak	Country	Likely source or vehicle	Year of outbreak	Healthcare Setting	Evidence: link cases and food vehicle	No of cases	No of deaths	Person-nel in cases	Age groups	Median (range) years	No of female cases/N	% females	Ref [a]
E. coli (ESBL)	Japan	Donor breast milk	2012	Neonatal intensive care unit	Epi analytical: case-control study; Mibi: detection in food vehicle or its component; detection in food chain or its environment	4	0	no	Newborn	(26-37 weeks)	3	50	[51]
E. coli O157	Japan	Pickled cabbage	2012	Nursing homes	Epi descriptive; detection in food vehicle or its component	107	5	no	Elderly	87 (72-102)	94	88	[52]
Campylobacter jejuni	Austria	Poultry dishes	2006	Tertiary care hospital	Epi descriptive	21	0	yes	Adults, Elderly?	NA	NA	NA	[53]
C. jejuni	Germany	Unknown	2015	Rehabilitation centre	No details/unknown/weak	15	0	NA	Adults, Elderly	62 (47-87)	10	67	SN27
C. jejuni	Germany	Unknown	2017	Rehabilitation centre	No details/unknown/weak	18	0	NA	Adults, Elderly	52 (23-65)	12	67	SN28
Group A ß-hemolytic streptococci (GAS)	Turkey	Milky dessert	2008	Hospital	Epi analytical: case-control study; Mibi: detection in food vehicle or its component	251	0	yes	Adults	NA	NA	NA	[54]
Klebsiella pneumoniae	Spain	Handmade fruit puree, soup	2008	Acute care Hospital	Epi analytical: case-control study	35	0	no[w]	Elderly	mean 69[x]	NA	43[x]	[55]
Citrobacter freundii (VIM Carbapenemase-Expressing)	Germany	Presliced vegetables	2016	University hospital	Epi descriptive; Mibi: detection in food vehicle or its component; detection in food chain or its environment	76[y]	0	no	Adults, Elderly	mean 69 (54-84)	43	45	[56]
Cyclospora cayetanensis	USA	Snow peas	2004	Residential facility	Epi analytical: cohort study; Product-tracing	96	0	NA	Adults	NA	NA	NA	[57, 58]
Blastoschizomyces capitatus	Spain	Milk	2002	Tertiary care hospital: haematology unit	Epi descriptive; Mibi: detection in food chain or its environment	4	2	no	Adults, Elderly	NA	NA	NA	[59]

[a] SN: SurvNet; Outbreak retrieved from the German electronic surveillance system (SurvNet@RKI) (search date: 11 September 2019)
SN*: Outbreaks categorised as "strong-evidence"-FBOs according to EFSA criteria [60]

[b] Mibi: microbiological evidence

[c] NA: not available

[d] Only for the 37 patients in HCFs (i.e. age and gender of 10 members of personnel not included)

[e] Based on 133 outbreak cases (i.e. including 31 visitors)

[f] Not clear if infant healthcare-associated

[g] Based on 41 outbreak cases (of which 29 cases were healthcare-associated)

[h] Case number reported to EFSA

[i] Infected food handler

[j] Based on 150 outbreak cases due to late reporting

[k] Norovirus: suspected aetiological agent, Kaplan criteria met

[l] Based on a total of 35 outbreak cases (i.e. 21 residents, eight staff members and six guests)

[m] Based on 204 outbreak cases (i.e. 114 patients and resident cases, 34 staff and 56 secondary cases)

[n] Risk factor: unprocessed contaminated ingredient, inadequate heat treatment

[o] Risk factor: unprocessed contaminated ingredient

[p] Information available for 18 outbreak cases

[q] Information available for 6 outbreak cases

[r] In total there were 57 outbreak cases (48 healthcare-associated, nine from community);
of 24 fatalities, not clear if all deaths were healthcare-associated

[s] Risk factor: Unprocessed contaminated ingredient

[t] For 15/20 case patients, the place of exposure was possibly in hospitals/long-term care

[u] For 8/9 deaths the place of exposure was possibly in hospitals/long-term care

[v] Based on 41 outbreaks cases (of which 29 considered healthcare-associated)

[w] 1 food handler and 5 cleaning staff were faecal carriers (not included in outbreak case number)

[x] Based on 156 colonized and/or infected patients

[y] Colonisations (i.e. not infections)

References

1. Hamada K & Tsuji H. Salmonella Brandenburg and S. Corvallis involved in a food poisoning outbreak in a hospital in Hyogo Prefecture. Jpn J Infect Dis 2001; 54(5):195-6. PMID: 11754160
2. Bruins MJ, Fernandes TM, Ruijs GJ, Wolfhagen MJ, van Rijn-van Berkel JM, Schenk BE, et al. Detection of a nosocomial outbreak of salmonellosis may be delayed by application of a protocol for rejection of stool cultures. J Hosp Infect 2003; 54(2):93-8. PMID: 12818580
3. Tribe IG, Cowell D, Cameron P & Cameron S. An outbreak of Salmonella typhimurium phage type 135 infection linked to the consumption of raw shell eggs in an aged care facility. Commun Dis Intell Q Rep 2002; 26(1):38-9. PMID: 11950200
4. Public Health Laboratory Services. Salmonella Enteritidis outbreak in a London hospital - update. CDR Wkly 2003; 13(8).
5. Miller T, Brockmann S, Spackova M, Wetzig J, Frank C, Pfeifer Y, et al. Recurrent outbreaks caused by the same Salmonella enterica serovar Infantis clone in a German rehabilitation oncology clinic from 2002 to 2009. J Hosp Infect 2018; 100(4):e233-e238. https://doi.org/10.1016/j.jhin.2018.03.035 PMID: 29614246
6. Elward A, Grim A, Schroeder P, Kieffer P, Sellenriek P, Ferrett R, et al. Outbreak of Salmonella javiana infection at a children's hospital. Infect Control Hosp Epidemiol 2006; 27(6):586-92. https://doi.org/10.1086/504935 PMID: 16755478
7. Heissenhuber A, Hautmann W, Arenz S, Kugler R, Kleih W, Ludwig S, et al. [Accumulated occurrence of illnesses with Salmonella enteritidis in hospitals and nursing homes in the district Oberallgaeu, Bavaria, in July 2004]. Gesundheitswesen 2005; 67(12):845-52. https://doi.org/10.1055/s-2005-858898 PMID: 16379046
8. Gikas A, Kritsotakis EI, Maraki S, Roumbelaki M, Babalis D, Scoulica E, et al. A nosocomial, foodborne outbreak of Salmonella Enterica serovar Enteritidis in a university hospital in Greece: the importance of establishing HACCP systems in hospital catering. J Hosp Infect 2007; 66(2):194-6. https://doi.org/10.1016/j.jhin.2007.03.001 PMID: 17482719
9. Frank C, Buchholz U, Maass M, Schroder A, Bracht KH, Domke PG, et al. Protracted outbreak of S. Enteritidis PT 21c in a large Hamburg nursing home. BMC Public Health 2007; 7:243. https://doi.org/10.1186/1471-2458-7-243 PMID: 17854497
10. Centers for Disease Control and Prevention. Salmonella Oranienburg infections associated with fruit salad served in health-care facilities--northeastern United States and Canada, 2006. MMWR Morb Mortal Wkly Rep 2007; 56(39):1025-8. PMID: 17914330
11. Jansen A, Hiller P, Desai S, Feier B, Habermann F, Baumann A, et al. [Protracted nosocomial outbreak of Salmonella Enteritidis LT 8/7]. Z Gastroenterol 2008; 46(11):1270-4. https://doi.org/10.1055/s-2008-1027543 PMID: 19012198
12. Poetter C, Kunstmann G, Peter D & Mattner F. Containment of a cheesecake-associated outbreak of salmonellosis in 3 different hospitals, detected by continuous microbiologic surveillance. Am J Infect Control 2014; 42(7):816-7. https://doi.org/10.1016/j.ajic.2014.03.013 PMID: 24825536
13. Bayer C, Bernard H, Prager R, Rabsch W, Hiller P, Malorny B, et al. An outbreak of *Salmonella* Newport associated with mung bean sprouts in Germany and the Netherlands, October to November 2011. Euro Surveill 2014; 19(1). PMID: 24434173
14. Styles T, Phan Q, Rabatsky-Ehr T, Applewhite C, Sosa L & Cartter M. Salmonella enterica serotype enteritidis outbreak at a long-term care facility, Connecticut, 2012. Conn Med 2013; 77(10):587-90. PMID: 24367841
15. Inns T, Lane C, Peters T, Dallman T, Chatt C, McFarland N, et al. A multi-country Salmonella Enteritidis phage type 14b outbreak associated with eggs from a German producer: 'near real-time' application of whole genome sequencing and food chain investigations, United Kingdom, May to September 2014. Euro Surveill 2015; 20(16). PMID: 25953273
16. Schielke A, Rabsch W, Prager R, Simon S, Fruth A, Helling R, et al. Two consecutive large outbreaks of Salmonella Muenchen linked to pig farming in Germany, 2013 to 2014: Is something missing in our regulatory framework? Euro Surveill 2017; 22(18). https://doi.org/10.2807/1560-7917.ES.2017.22.18.30528 PMID: 28494842
17. Almagro Nievas D, Conti Cuesta F, Espinola Garcia E, Morcillo Rodenas C, Nunez Sevilla C, Linares Torres J, et al. [Outbreak of gastroenteritis caused by Norwalk virus at a senior citizens assisted living facility in Granada, Spain]. Rev Esp Salud Publica 2003; 77(2):283-91. PMID: 12728663
18. Eriksen HM, Guerin PJ, Nygard K, Hjertqvist M, de Jong B, Rose AM, et al. Gastro-enteritis outbreak among Nordic patients with psoriasis in a health centre in Gran Canaria, Spain: a cohort study. BMC Infect Dis 2004; 4:45. https://doi.org/10.1186/1471-2334-4-45 PMID: 15511300
19. Falkenhorst G, Krusell L, Lisby M, Madsen SB, Bottiger B & Molbak K. Imported frozen raspberries cause a series of norovirus outbreaks in Denmark, 2005. Euro Surveill 2005; 10(9):E050922 2. PMID: 16788235

20. Medici MC, Morelli A, Arcangeletti MC, Calderaro A, De Conto F, Martinelli M, et al. An outbreak of norovirus infection in an Italian residential-care facility for the elderly. Clin Microbiol Infect 2009; 15(1):97-100. https://doi.org/10.1111/j.1469-0691.2008.02117.x PMID: 19220341
21. Ohwaki K, Nagashima H, Aoki M, Aoki H & Yano E. A foodborne norovirus outbreak at a hospital and an attached long-term care facility. Jpn J Infect Dis 2009; 62(6):450-4. PMID: 19934537
22. Schmid D, Kuo HW, Hell M, Kasper S, Lederer I, Mikula C, et al. Foodborne gastroenteritis outbreak in an Austrian healthcare facility caused by asymptomatic, norovirus-excreting kitchen staff. J Hosp Infect 2011; 77(3):237-41. https://doi.org/10.1016/j.jhin.2010.11.015 PMID: 21272956
23. Hoffmann D, Mauroy A, Seebach J, Simon V, Wantia N & Protzer U. New norovirus classified as a recombinant GII.g/GII.1 causes an extended foodborne outbreak at a university hospital in Munich. J Clin Virol 2013; 58(1):24-30. https://doi.org/10.1016/j.jcv.2013.06.018 PMID: 23849648
24. Bernard H, Faber M, Wilking H, Haller S, Hohle M, Schielke A, et al. Large multistate outbreak of norovirus gastroenteritis associated with frozen strawberries, Germany, 2012. Euro Surveill 2014; 19(8):20719. PMID: 24602278
25. Loury P, Le Guyader FS, Le Saux JC, Ambert-Balay K, Parrot P & Hubert B. A norovirus oyster-related outbreak in a nursing home in France, January 2012. Epidemiol Infect 2015; 143(12):2486-93. https://doi.org/10.1017/s0950268814003628 PMID: 25567093
26. Shetty A, McLauchlin J, Grant K, O'Brien D, Howard T & Davies EM. Outbreak of Listeria monocytogenes in an oncology unit associated with sandwiches consumed in hospital. J Hosp Infect 2009; 72(4):332-6. https://doi.org/10.1016/j.jhin.2009.01.012 PMID: 19278752
27. Dawson SJ, Evans MR, Willby D, Bardwell J, Chamberlain N & Lewis DA. Listeria outbreak associated with sandwich consumption from a hospital retail shop, United Kingdom. Euro Surveill 2006; 11(6):89-91. PMID: 16801694
28. Health Protection Agency. Human listeriosis linked to hospital sandwiches: implications for procurement and storage. Health Protection Report 2(35). 29 August 2008 news. 2008. https://webarchive.nationalarchives.gov.uk/20140714101922/http://www.hpa.org.uk/hpr/archives/2008/news3508.htm (accessed 29.03.2020).
29. Winter CH, Brockmann SO, Sonnentag SR, Schaupp T, Prager R, Hof H, et al. Prolonged hospital and community-based listeriosis outbreak caused by ready-to-eat scalded sausages. J Hosp Infect 2009; 73(2):121-8. https://doi.org/10.1016/j.jhin.2009.06.011 PMID: 19716628
30. Johnsen BO, Lingaas E, Torfoss D, Strom EH & Nordoy I. A large outbreak of Listeria monocytogenes infection with short incubation period in a tertiary care hospital. J Infect 2010; 61(6):465-70. https://doi.org/10.1016/j.jinf.2010.08.007 PMID: 20813130
31. Currie A, Farber JM, Nadon C, Sharma D, Whitfield Y, Gaulin C, et al. Multi-province listeriosis outbreak linked to contaminated deli meat consumed primarily in institutional settings, Canada, 2008. Foodborne Pathog Dis 2015; 12(8):645-52. https://doi.org/10.1089/fpd.2015.1939 PMID: 26258258
32. Public Health Agency Northern Ireland. The report of the Outbreak Control Team of the investigation of an outbreak of listeriosis in the Belfast Health and Social Care Trust during May to November 2008. 2009. https://www.publichealth.hscni.net/sites/default/files/ListeriaReport.pdf (accessed 29.03.2020).
33. Cokes C, France AM, Reddy V, Hanson H, Lee L, Kornstein L, et al. Serving high-risk foods in a high-risk setting: survey of hospital food service practices after an outbreak of listeriosis in a hospital. Infect Control Hosp Epidemiol 2011; 32(4):380-6. https://doi.org/10.1086/658943 PMID: 21460490
34. Little CL, Gormley F, Mook P, Gillespie I, Foster K, Harris J, et al. Advisory Committee on the Microbiological Safety of Food Information Paper. Outbreaks of infection associated with ready-to-eat foods. ACM/1014. London: Food Standards Agency. 2011. https://acmsf.food.gov.uk/sites/default/files/mnt/drupal_data/sources/files/multimedia/pdfs/committee/acm1014hpa.pdf.
35. Gaul LK, Farag NH, Shim T, Kingsley MA, Silk BJ & Hyytia-Trees E. Hospital-acquired listeriosis outbreak caused by contaminated diced celery--Texas, 2010. Clin Infect Dis 2013; 56(1):20-6. https://doi.org/10.1093/cid/cis817 PMID: 22997210
36. Centers for Disease Control and Prevention. (2015). Multistate outbreak of listeriosis linked to Blue Bell creameries products (Final Update). https://www.cdc.gov/listeria/outbreaks/ice-cream-03-15/index.html
37. Coetzee N, Laza-Stanca V, Orendi JM, Harvey S, Elviss NC & Grant KA. A cluster of Listeria monocytogenes infections in hospitalised adults, Midlands, England, February 2011. Euro Surveill 2011; 16(20):19869. PMID: 21616050
38. Jacks A, Pihlajasaari A, Vahe M, Myntti A, Kaukoranta SS, Elomaa N, et al. Outbreak of hospital-acquired gastroenteritis and invasive infection caused by Listeria monocytogenes, Finland, 2012. Epidemiol Infect 2016; 144(13):2732-42. https://doi.org/10.1017/s0950268815002563 PMID: 26493730
39. Najjar Z, Gupta L, Sintchenko V, Shadbolt C, Wang Q & Bansal N. Listeriosis cluster in Sydney linked to hospital food. Med J Aust 2015; 202(8):448-9. PMID: 25929511
40. Kvistholm Jensen A, Nielsen EM, Bjorkman JT, Jensen T, Muller L, Persson S, et al. Whole-genome sequencing used to investigate a nationwide outbreak of listeriosis caused by ready-to-eat delicatessen meat, Denmark, 2014. Clin Infect Dis 2016; 63(1):64-70. https://doi.org/10.1093/cid/ciw192 PMID: 27025820

41. Rietberg K, Lloyd J, Melius B, Wyman P, Treadwell R, Olson G, et al. Outbreak of Listeria monocytogenes infections linked to a pasteurized ice cream product served to hospitalized patients. Epidemiol Infect 2016; 144(13):2728-31. https://doi.org/10.1017/s0950268815003039 PMID: 27586030
42. Mazengia E, Kawakami V, Rietberg K, Kay M, Wyman P, Skilton C, et al. Hospital-acquired listeriosis linked to a persistently contaminated milkshake machine. Epidemiol Infect 2017; 145(5):857-863. https://doi.org/10.1017/S0950268816003198 PMID: 28065212
43. Li Z, Perez-Osorio A, Wang Y, Eckmann K, Glover WA, Allard MW, et al. Whole genome sequencing analyses of Listeria monocytogenes that persisted in a milkshake machine for a year and caused illnesses in Washington State. BMC Microbiol 2017; 17(1):134. https://doi.org/10.1186/s12866-017-1043-1 PMID: 28619007
44. Decousser JW, Ramarao N, Duport C, Dorval M, Bourgeois-Nicolaos N, Guinebretiere MH, et al. Bacillus cereus and severe intestinal infections in preterm neonates: Putative role of pooled breast milk. Am J Infect Control 2013; 41(10):918-21. https://doi.org/10.1016/j.ajic.2013.01.043 PMID: 23769834
45. Schmid D, Rademacher C, Kanitz EE, Frenzel E, Simons E, Allerberger F, et al. Elucidation of enterotoxigenic Bacillus cereus outbreaks in Austria by complementary epidemiological and microbiological investigations, 2013. Int J Food Microbiol 2016; 232:80-6. https://doi.org/10.1016/j.ijfoodmicro.2016.05.011 PMID: 27257745
46. Tanaka D, Isobe J, Hosorogi S, Kimata K, Shimizu M, Katori K, et al. An outbreak of food-borne gastroenteritis caused by *Clostridium perfringens* carrying the cpe gene on a plasmid. Jpn J Infect Dis 2003; 56(3):137-9. PMID: 12944687
47. Bos J, Smithee L, McClane B, Distefano RF, Uzal F, Songer JG, et al. Fatal necrotizing colitis following a foodborne outbreak of enterotoxigenic *Clostridium perfringens* type A infection. Clin Infect Dis 2005; 40(10):e78-83. https://doi.org/10.1086/429829 PMID: 15844055
48. Centers for Disease Control and Prevention. Fatal foodborne Clostridium perfringens illness at a state psychiatric hospital--Louisiana, 2010. MMWR Morb Mortal Wkly Rep 2012; 61(32):605-8. PMID: 22895383
49. Bolduc D, Srour LF, Sweet L, Neatby A, Galanis E, Isaacs S, et al. Severe outbreak of Escherichia coli O157:H7 in health care institutions in Charlottetown, Prince Edward Island, fall, 2002. Can Commun Dis Rep 2004; 30(9):81-8. PMID: 15132397
50. Reiss G, Kunz P, Koin D & Keeffe EB. Escherichia coli O157:H7 infection in nursing homes: review of literature and report of recent outbreak. J Am Geriatr Soc 2006; 54(4):680-4. https://doi.org/10.1111/j.1532-5415.2006.00682.x PMID: 16686882
51. Nakamura K, Kaneko M, Abe Y, Yamamoto N, Mori H, Yoshida A, et al. Outbreak of extended-spectrum beta-lactamase-producing Escherichia coli transmitted through breast milk sharing in a neonatal intensive care unit. J Hosp Infect 2016; 92(1):42-6. https://doi.org/10.1016/j.jhin.2015.05.002 PMID: 26238662
52. Tabuchi A, Wakui T, Yahata Y, Yano K, Azuma K, Yamagishi T, et al. A large outbreak of enterohaemorrhagic Escherichia coli O157, caused by low-salt pickled Napa cabbage in nursing homes, Japan, 2012. Western Pac Surveill Response J 2015; 6(2):7-11. https://doi.org/10.5365/wpsar.2014.5.1.012 PMID: 26306209
53. Jelovcan S, Schmid D, Lederer I, Hell M, Rehberger K, Arnhold D, et al. Cluster of nosocomial campylobacteriosis, Austria 2006. J Hosp Infect 2008; 69(1):97-8. https://doi.org/10.1016/j.jhin.2008.01.010 PMID: 18329135
54. Ertugrul BM, Erol N, Emek M, Ozturk B, Saylak OM, Cetin K, et al. Food-borne tonsillopharyngitis outbreak in a hospital cafeteria. Infection 2012; 40(1):49-55. https://doi.org/10.1007/s15010-011-0166-9 PMID: 21826437
55. Calbo E, Freixas N, Xercavins M, Riera M, Nicolas C, Monistrol O, et al. Foodborne nosocomial outbreak of SHV1 and CTX-M-15-producing Klebsiella pneumoniae: epidemiology and control. Clin Infect Dis 2011; 52(6):743-9. https://doi.org/10.1093/cid/ciq238 PMID: 21367727
56. Pletz MW, Wollny A, Dobermann UH, Rodel J, Neubauer S, Stein C, et al. A nosocomial foodborne outbreak of a VIM Carbapenemase-expressing Citrobacter freundii. Clin Infect Dis 2018; 67(1):58-64. https://doi.org/10.1093/cid/ciy034 PMID: 29346622
57. Centers for Disease Control and Prevention. Outbreak of cyclosporiasis associated with snow peas--Pennsylvania, 2004. MMWR Morb Mortal Wkly Rep 2004; 53(37):876-8. PMID: 15385921
58. Heilpern KL & Wald M. Update on emerging infections: news from the Centers for Disease Control and Prevention. Outbreak of cyclosporiasis associated with snow peas--Pennsylvania, 2004. Ann Emerg Med 2005; 45(5):529-31. https://doi.org/10.1016/j.annemergmed.2005.02.006 PMID: 15855952
59. Gurgui M, Sanchez F, March F, Lopez-Contreras J, Martino R, Cotura A, et al. Nosocomial outbreak of Blastoschizomyces capitatus associated with contaminated milk in a haematological unit. J Hosp Infect 2011; 78(4):274-8. https://doi.org/10.1016/j.jhin.2011.01.027 PMID: 21658800
60. EFSA (European Food Safety Authority). Update of the technical specifications for harmonised reporting of food-borne outbreaks through the European Union reporting system in accordance with Directive 2003/99/EC. EFSA Journal 2014; 12((3):3598). https://doi.org/10.2903/j.efsa.2014.3598
